# Obstetric Complications and Psychological Well-being: Experiences of Bangladeshi Women during Pregnancy and Childbirth

**DOI:** 10.3329/jhpn.v30i2.11310

**Published:** 2012-06

**Authors:** K. Gausia, D. Ryder, M. Ali, C. Fisher, A. Moran, M. Koblinsky

**Affiliations:** ^1^icddr,b, GPO Box 128, Dhaka 1000, Bangladesh; ^2^School of Exercise, Biomedical and Health Sciences, Edith Cowan University, Joondalup, Western Australia; ^3^Combined Universities Centre for Rural Health, University of Western Australia, Perth, Australia; ^4^Centre for International Health, Curtin University of Technology, Perth, Australia; ^5^University of Western Australia, Perth, Australia; ^6^Save the Children Fund-USA, Washington, DC, USA; ^7^John Snow Inc., Arlington, Virginia 22205, USA

**Keywords:** Childbirth, Delivery, Depression, Obstetric complications, Pregnancy, Bangladesh

## Abstract

Women in developing countries experience postnatal depression at rates that are comparable with or higher than those in developed countries. However, their personal experiences during pregnancy and childbirth have received little attention in relation to postnatal depression. In particular, the contribution of obstetric complications to their emotional well-being during the postpartum period is still not clearly understood. This study aimed to (a) describe the pregnancy and childbirth experiences among women in Bangladesh during normal childbirth or obstetric complications and (b) examine the relationship between these experiences and their psychological well-being during the postpartum period. Two groups of women—one group with obstetric complications (n=173) and the other with no obstetric complications (n=373)—were selected from a sample of women enrolled in a community-based study in Matlab, Bangladesh. The experiences during pregnancy and childbirth were assessed in terms of a five-point rating scale from ‘severely uncomfortable=1’ to ‘not uncomfortable at all=5’. The psychological status of the women was assessed using a validated local version of the Edinburgh Postnatal Depression Scale (EPDS) at six weeks postpartum. Categorical data were analyzed using the chi-square test and continuous data by analysis of variance. Women with obstetric complications reported significantly more negative experiences during their recent childbirth [95% confidence interval (CI) 1.36-1.61, p<0.001] compared to those with normal childbirth. There was a significant main effect on emotional well-being due to experiences of pregnancy [F (4,536)=4.96, p=0.001] and experiences of childbirth [F (4,536)=3.29, p=0.01]. The EPDS mean scores for women reporting severe uncomfortable pregnancy and childbirth experiences were significantly higher than those reporting no such problems. After controlling for the background characteristics, postpartum depression was significantly associated with women reporting a negative childbirth experience. Childbirth experiences of women can provide important information on possible cases of postnatal depression.

## INTRODUCTION

While childbirth is a positive experience for many women, it can be a traumatic one for others (1). Studies examining childbirth experiences of women have predominantly focused on various aspects of labour pain (2-4), control in decision-making in relation to delivery procedures, and satisfaction with overall delivery circumstances, including satisfaction with services received during labour (5-9). A few studies have examined the impact of pregnancy and childbirth experiences of women on psychological well-being, particularly their risks of postnatal depression—a major public-health problem worldwide.

Women in developing countries experience postnatal depression at rates that are comparable with or higher than those in developed countries (10). One-quarter of Asian women suffer from depression after childbirth. Surveys have found a 23% prevalence of postnatal depression in India (11), 28% in Pakistan (12), and 22% in Bangladesh (13). While the aetio-logy of postnatal depression is complex and multifaceted, the psychosocial factors have been identified as strong predictors for postnatal depression globally. Additionally, obstetric complications and delivery circumstances can be significant stressors for women and have been postulated as risk factors for postnatal depression. However, findings from the large number of studies examining the association between obstetric complications and postnatal depression are ambiguous (14-19).

Although some studies have demonstrated significantly higher levels of postnatal depression among women who experienced obstetric complications (14-16), others have failed to prove such links (17-19). For example, a prospective study on 490 women in New South Wales, Australia, did not find any significant association between complications during pregnancy and labour and postnatal depression (17). Another prospective study in France found a link between severe antepartum complications and increased severity of postnatal depression at six weeks postpartum but no such links could be found for complications during labour or delivery (18). Similar results have been reported from Manchester, England (19). All the three studies used medical definitions and diagnosis of obstetric complications, such as prolonged labour, eclampsia, antepartum haemorrhage, gestational diabetes, third-degree tear, etc. and ignored the women's self-reported personal experiences of childbirth.

Evidence suggests that pregnancy and childbirth experiences impact on the psychological state of women in the postpartum period (7). A prospective study on 825 women in southeast England found that measures of feelings of discomfort during pregnancy and childbirth were significantly associated with low psychological well-being in the postpartum period. Their subjective experience of childbirth appeared to have a greater impact on psychological state than other more objective aspects of childbirth, such as instrumentation or clinical interventions during delivery, e.g. episiotomies, enemas, and forceps deliveries.

We found no published literature in Bangladesh that directly examined the childbirth experiences of women and their psychological status in the postpartum period. However, the little that has been reported looked at issues, such as how women distinguished between normal and complicated birth, what elements are required to achieve a normal birth, and the various sociocultural aspects of childbirth (20). This ethnographic study on 100 rural women found that Bangladeshi women re-cognized the need to have both physical strength (*shoriler shakti*) and mental courage (*moner shahosh*) to survive the childbirth experience, indicating the psychological aspects of childbirth (20). The present study aimed to (a) describe the pregnancy and childbirth experiences among women in Bangladesh during normal childbirth or obstetric complications and (b) examine the relationship between these experiences and their psychological well-being during the postpartum period.

## MATERIALS AND METHODS

**Fig. 1.**
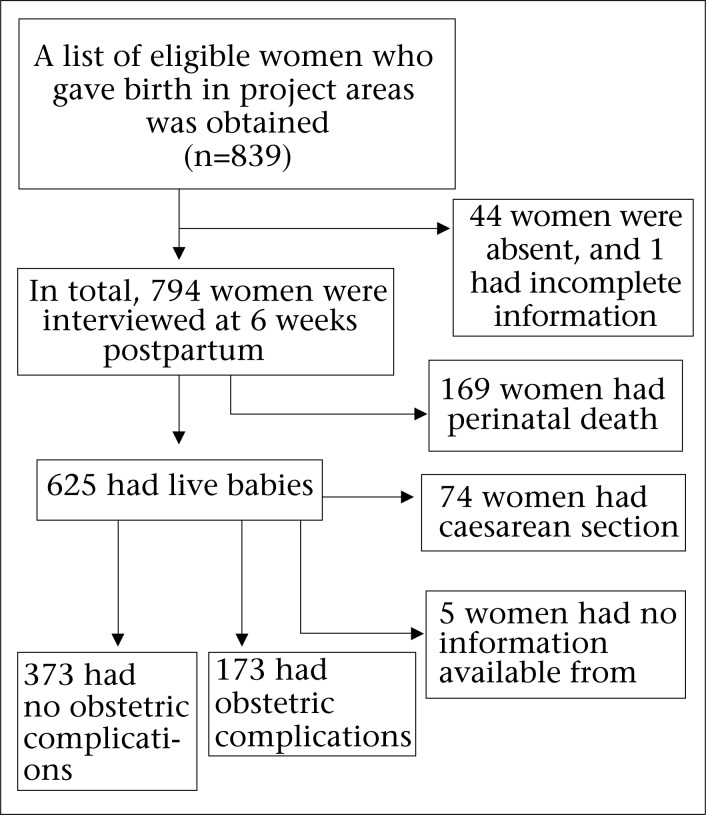
Pattern of recruitment of study participants

This study was part of a larger community-based study examining the burden of maternal ill-health and its physical, psychological, social and economic consequences among women in Matlab, a rural subdistrict in eastern Bangladesh. Here we describe a subsample of women who gave births during January 2007–March 2008 in the Matlab Maternal and Child Health–Family Planning (MCH-FP) project area of the International Centre for Diarrhoeal Disease Research, Bangladesh (icddr,b). The pattern of recruitment of the study participants is shown in Figure 1.

### Sample

During the study period, the Community Health Research Workers (CHRWs) documented the place of birth (e.g. home-delivery, delivery at health facili-ty) and outcome (e.g. stillbirth, livebirth) of recent deliveries in the project area on a fortnightly basis. Two study physicians reviewed medical records using a structured data-collection form to assess obs-tetric complications among women who delivered in health facilities. The list of recently-delivering women was updated on continuous basis as soon as the CHRWs collected delivery-related information. Women who had a livebirth and satisfied the selection criteria were included in the study. Cases of normal childbirth were randomly selected (half of them were home-deliveries and the other half at health facilities) from among women who gave births in the Matlab MCH-FP area during January 2007–March 2008.

### Selection criteria

Cases of obstetric complications—both severe and less severe—were identified by reviewing the medical records at the Matlab Hospital and emergency obstetric facilities in Chandpur district and were included in the study in an ongoing process. Operational definitions for severe maternal complications encompassed cases of severe dystocia, severe haemorrhage, eclampsia or severe pre-eclampsia, septic shock, septicaemia, and severe anaemia while less-severe maternal complications included vaginal bleeding, pre-eclampsia, dystocia, infection, or non-severe anaemia (Koblinsky M. Personal communication, 2005).

Singleton, cephalic presentation and full-term (gestation of 37 weeks or more) normal vaginal delivery either at home or at health facilities were considered normal vaginal delivery. Women with perinatal deaths and hospitalization for postpartum complications within two weeks after normal vaginal delivery did not qualify to be in the group of women with normal deliveries.

### Data-collection

Several sets of questionnaire, including delivery-record questionnaire, physical examination questionnaire, and mental health questionnaire, were used for collecting a range of information from the study participants. For this analysis, we emphasized two specific questions regarding the overall experiences of the study participants during pregnancy and childbirth. Trained female interviewers collected information from the selected study participants at their home at six weeks postpartum using the structured questionnaire. The questionnaire started with sociodemographic questions. Then the experiences of pregnancy and childbirth were assessed in terms of a five-point rating scale ranging from ‘severely uncomfortable=1’ to ‘not uncomfortable at all=5’. The exact question that was asked to collect information on their self-reported experiences of pregnancy was “How do you rate your experience during the last pregnancy?” For childbirth, the question asked was “How do you rate your experience during your recent delivery?” In the same setting, the psychological status of the study participants was assessed using a validated local version of the Edinburgh Postnatal Depression Scale, called EPDS-B (21).

The EPDS is an internationally-accepted and widely-validated tool used for assessing the depression status among women in the postpartum period (22). The EPDS was translated into Bangla and validated for use by local healthcare workers in Bangladesh (21,23). A cut-off score of 10 or above on the EPDS-B scale was found to be optimum in ascertaining depression among postnatal women.

### Analysis of data

Data were analyzed using the SPSS software (version 18). For the categorical variables, the chi-square test and, for the continuous variables, the Independent sample *t*-test and two-way analysis of variance (ANOVA) were applied. Binary logistic regression analysis was also performed to isolate contributory factors for postpartum depression. The background variables that were significantly (p<0.05) different between the two childbirth groups (normal and with obstetric complications), negative experiences of pregnancy, negative experiences of childbirth, and women with and without obstetric complications were entered into the logistic regression model. The questions specifically addressed in this analysis were: Are there any differences of emotional well-being among the two groups of the study participants—one with obstetric complications and the other with normal childbirth? Is there any impact of the self-reported experiences of pregnancy/childbirth and of birth groups (obstetric complications vs normal childbirth) on emotional well-being in the postpartum period?

### Ethical approval

The ethics and research review committees of icddr,b approved the project. Informed consent was obtained from the participants before interviewing them. Appropriate medical (obstetric) and psychological (higher EPDS-B scores) services were provided to the participants if they were thought to need such services during the study duration.

## RESULTS

In total, 794 study participants were assessed for both obstetric and psychological information (Fig. 1). Of them, 169 were excluded because they experienced a perinatal death, 74 had a caesarean section without an absolute maternal indication, and five were excluded due to lack of information about the status of their obstetric complications in their hospital-records. Therefore, final analysis was carried out on 546 study participants who had complete obstetric and psychological information on assessment at six weeks after childbirth. Of the 546 participants, 73 (13.4%) had very severe maternal complications, 100 (18.3%) had less-severe maternal complications, and 373 (68.3%) had normal vaginal delivery. Given the small numbers, groups of very severe and less-severe maternal complications were pooled and re-categorized as the obstetric complications group (n=173) and compared with the normal childbirth group (n=373).

### Sociodemographic characteristics

The ages of the 546 study participants ranged from 13 years to 45 years, with a mean age of 26.13 years and standard deviation (SD) of 5.95 years. Around 15% had no formal education, 27.3% had primary education, and only 6.6% had education beyond high school (10 years). Most (80.2%) participants were Muslim, 10.4% were Hindu, and from the remaining 9.3%, we had no information available on their religious affiliation. Most (99.6%) study participants were in marital relationships; only two were living separately from their husbands during assessment at six weeks postpartum. About one-third (30.4%) were primipara, and the remaining were multipara; 9.5% of the participants had unknown parity. The large majority (79.1%) were housewives. A very few (3.8%) women had paid jobs.

Table 1 shows the selected basic characteristics of the study participants with and without obstetric complications. No significant differences were found on the basic characteristics among the study participants with and without obstetric complications, except regarding education, parity, and economic status. Higher education (>10 years of schooling), primiparity, and belonging to the richest socioeconomic quintile were significantly linked to the obstetric complications group. Of the 546 participants, we had no information from 9.3% on the gender of their children. For the remainder, the distribution of births of male and female children was approximately equal (45.2% and 45.5% respectively).

Table 2 shows that 31.8% of the 173 participants of the obstetric complications group reported that their recent pregnancy was severely uncomfortable. However, 27.2% of the participants also mentioned that they were not uncomfortable at all during pregnancy. On the other hand, 33.8% of the participants in the normal childbirth group reported that their recent pregnancy was not uncomfortable, and they had not had any problem at all. Less than one-fifth (17.7%) in the normal childbirth group felt that their recent pregnancy was severely uncomfortable or unpleasant (Table 2).

Enquiry into their self-reported experiences of childbirth revealed that, overall, 37.4% of the participants reported a severely-uncomfortable experience with their recent childbirth while 26.2% felt that it was not uncomfortable at all (Table 3). Not surprisingly, 71.1% of the participants in the obstetric complications group found their last childbirth experience to be severely uncomfortable compared to 21.7% in the normal childbirth group (Table 3).

Table 4 summarizes the self-reported experiences during pregnancy and childbirth, which shows that these differed significantly between women experiencing obstetric complications compared to those with normal childbirth. Participants reporting any level of discomfort during pregnancy were recoded as negative experiences of pregnancy. Similarly, participants with any level of discomfort during childbirth were recoded as negative experience of childbirth. The participants of the obstetric complications group reported significantly more negative experiences during their recent childbirth [95% confidence interval (CI) 1.36-1.61, p<0.001] compared to those with normal childbirth (Table 4).

### Psychological outcomes

Women with obstetric complications had a mean EPDS score of 4.40 [standard deviation (SD)±4.37] compared to 5.08 (SD±4.77) for women with normal childbirth. This was not significant as determined by an independent sample *t*-test. Based on the total EPDS cut-off scores, the rate of depression symptoms was 12.1% (n=21) in the obstetric complications group and 16.9% (n=63) in the normal childbirth group, and the differences were not significant. However, women with self-reported negative experiences (uncomfortable) of pregnancy had a significantly higher rate of depression than women who reported a normal pregnancy (18.0% vs 9.8%, p<0.02). Similarly, women who were uncomfortable at any level of childbirth were more depressed than women with normal childbirth (17.9% vs 8.2%, p<0.01).

**Fig. 2.**
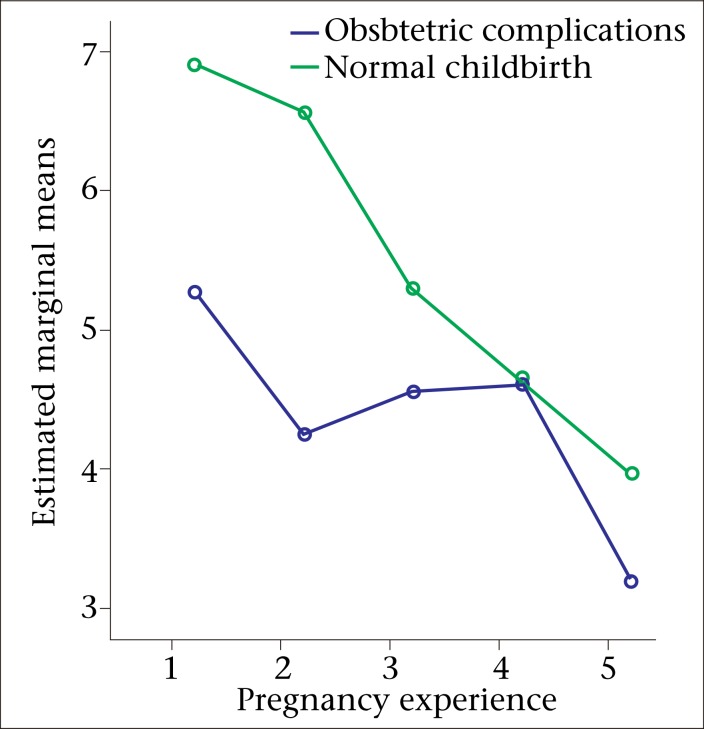
Relationship between perceived levels of uncomfortability of pregnancy and postpartum emotional well-being among mothers in Matlab, Bangladesh

To examine the impact of the birthing groups (e.g. obstetric complications group and normal childbirth group) and pregnancy experiences on the level of depression as measured by the EPDS, a two-way between-groups ANOVA was conducted (Fig. 2). The study participants were divided into five groups according to their self-reported pregnancy experiences (1=severely uncomfortable to 5=not uncomfortable at all). There was a significant main effect for self-reported experiences of any level of uncomfort during pregnancy [F (4,536)=4.96, p=0.001]; however, the effect-size was small (partial eta squared=0.04). The post-hoc comparison indicated that the mean score for women who said that they had a ‘severely-uncomfortable’ pregnancy (M=6.17, SD±5.45) was significantly different from women who said that their pregnancy was ‘not uncomfortable at all’ (M=3.76, SD±4.18). The remaining three groups did not differ significantly from one another. The main effect for the birthing groups [F (1,536)=4.10, p=0.04] and the interaction effect [F (4,536)=0.45, p=0.77] did not reach statistical significance.

**Table 1.** Background characteristics of study women

Characteristics	Birthing group				Significance tests p value
	Obstetric complications		Normal childbirth		
	No. (n=173)	%	No. (n=373)	%	
Age (years)					
<20	26	15.0	48	12.9	χ^2^=1.49; p=0.48
20-34	132	76.3	281	75.3	
>34	15	8.7	44	11.8		
Education					
No education	16	9.2	66	17.7	χ^2^=41.57; p<0.001
Primary (1-5 years)	30	17.3	119	31.9	
Secondary (6-10 years)	102	59.0	177	45.5	
Above secondary	25	14.5	11	2.9	
Religion					
Muslim	132	76.3	306	82.0	χ^2^=3.41; p=0.18
Hindu	24	13.9	33	8.8	
Unknown	17	9.8	34	9.1		
Marital status					
Currently married	172	99.4	372	99.7	χ^2^=3.1; p=<0.53†
Separated/widowed	1	0.6	1	0.3		
Parity					
Primipara	77	44.5	89	23.9
Multipara	78	23.8	250	67.0	χ^2^=26.25; p<0.001
Unknown	18	10.4	34	9.1	
Occupation					
Household work	146	84.4	323	86.6	χ^2^=0.51; p=0.77
Paid job	7	4.0	14	3.8	
Unknown	20	11.6	36	9.7		
Economic status*					
Poorest	11	6.4	74	19.8	χ^2^=53.23; p<0.001
Less poor	24	13.9	61	16.4	
Middle	19	11.0	77	20.6	
Richer	26	15.0	66	17.7		
Richest	78	45.1	68	18.2		
Unknown	15	8.9	27	7.2		

*Asset quintile

†Fisher's exact text

**Table 2.** Experience concerning recent pregnancy period

Experiences during pregnancy	Birthing group				All	
	Obstetric complications (n=173)		Normal childbirth (n=373)			
	No.	%	No.	%	No.	%
Severely uncomfortable	55	31.8	66	17.7	121	22.2
Moderately uncomfortable	8	4.6	16	4.3	24	4.4
Uncomfortable	45	26.0	101	27.1	146	26.7
Mildly uncomfortable	18	10.4	64	17.2	82	15.0
Not uncomfortable at all	47	27.2	126	33.8	173	31.7

**Table 3.** Experience concerning recent childbirth

Experiences during childbirth	Birthing group				All	
	Obstetric complications (n=173)		Normal childbirth (n=373)			
	No.	%	No.	%	No.	%
Severely uncomfortable	123	71.1	81	21.7	204	37.4
Moderately uncomfortable	11	6.4	11	2.9	22	4.0
Uncomfortable	26	15.0	93	24.9	119	21.8
Mildly uncomfortable	4	2.3	54	14.5	58	10.6
Not uncomfortable at all	9	5.2	134	35.9	143	26.2

**Table 4.** Negative experience concerning recent pregnancy and childbirth

Perceived negative experiences	Birthing group						p value (95% CI)
	Obstetric complications (n=173)		Normal childbirth (n=373)		Total		
	No.	%	No.	%	No.	%	
Pregnancy							
Yes	126	33.8	247	66.2	373	100	0.15
No	47	27.2	126	72.2	173	100	(0.98-1.23)
Childbirth							
Yes	164	40.7	239	59.3	403	100	<0.001
No	9	6.3	134	93.7	143	(1.36-1.61)

CI=Confidence interval

**Fig. 3.**
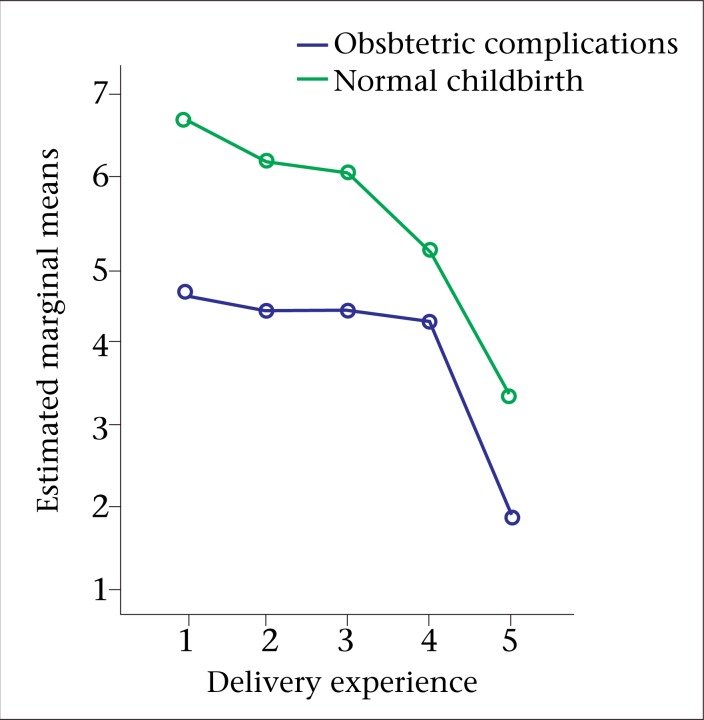
Relationship between perceived levels of uncomfortability of childbirth and postpartum emotional well-being among mothers in Matlab, Bangladesh

Similarly, the impact of the birthing groups on their recent childbirth experiences was analyzed. The study participants were also divided into five groups according to their childbirth experiences (1=severely uncomfortable to 5=not uncomfortable at all) on the level of depression (Fig. 3). There was a significant main effect for experiences of childbirth [F (4,536)=3.29, p=0.01]; however, the effect-size was small (partial eta squared=0.02). The post-hoc comparison indicated that the mean score for the women who mentioned that they had a severely-uncomfortable childbirth (M=5.43, SD±4.81) was significantly different from that for the women who mentioned that their deliveries were not uncomfortable at all (M=3.24, SD±3.88). The remaining three groups did not differ significantly from one another. The main effect of birthing groups [F (1,536)=4.73, p=0.03] and the interaction effect [F (4,536)=0.11, p=0.98] did not reach statistical significance.

Results of logistic regression analysis indicated that self-reported negative childbirth experience of women was independently associated with postpartum depression. After controlling for background characteristics (educational level, parity, and economic status), postpartum depression was significantly associated with women reporting a negative childbirth experience. The odds were three times higher (adjusted odds ratio=3.11, p=0.002, CI 1.52-6.36) for women with negative childbirth experiences to become depressed at postpartum than those with no negative childbirth experiences. No other variables were significantly associated with postpartum depression.

## DISCUSSION

This study found a significant association between women's self-reported negative experience of pregnancy and childbirth (meaning any level of discomfort) and subsequent postpartum depression. Not surprisingly, medically-defined obstetric complications were associated with a negative subjective experience of childbirth. However, obstetric complications were not significantly associated with negative experiences of pregnancy itself or with postnatal depression.

Women's negative experiences of pregnancy and childbirth were significantly associated with depression status during the postpartum period in this study. Green *et al*. also found that women in southeast England, who had negative experiences of their pregnancy or uncomfortable childbirth, had low emotional well-being (7). Their study distinguished between subjective experiences of how the woman felt during labour and the objective events resulting from obstetric procedures, such as episiotomy, enema, forceps delivery, and caesarean sections. Interestingly, these obstetric procedures, except caesarean section, did not substantially affect the emotional well-being of women, although they did detract from their overall satisfaction level with childbirth experiences (7). Thus, it seems that the subjective experience of childbirth is important from a psychological point of view, although it is difficult to measure or quantify precisely. One reason could be that individuals use their own invisi-ble personal yardstick regarding their experiences of pregnancy and childbirth (8). Additionally, a recent review found that the experience of childbirth is a complex and highly-subjective process that incorporates interrelated subjective psychological and physiological aspects that are influenced by the social, environmental, organizational and policy contexts (24).

In the present study, women with severely-uncomfortable experiences of pregnancy and childbirth had significantly higher mean EPDS scores than women who had not had an uncomfortable pregnancy or childbirth. Although we did not ask women about the available support mechanisms they resorted to, results of studies showed that lack of support both during pregnancy and after childbirth are significantly associated with postnatal depression (25-27). A population-based case-control study found that Australian women who had been depressed after birth reported receiving less practical help (with household tasks, for example) and emotional support from their partners and saw themselves as having less social support overall than women in the control group (27). Certainly, more research is needed to clearly understand the various support elements and their contributions to the emotional well-being of mothers in Bangladesh.

Although, in this study, higher education and socio-economic status were not linked to the depression status of women, their association with obstetric complications could have been due to better care-seeking among this group. Our study did not find a significant difference on rates of depression among women with obstetric complications and women having normal childbirth. This finding is consistent with results of studies conducted elsewhere (17-19). Studies that documented higher rates of depression among women with obstetric complications used different methodologies. For example, complications included perinatal variables, such as suspected foetal distress, hospitalization of the newborn baby, and perinatal deaths (15,16).

Women with negative experiences of childbirth in our study also tended to have obstetric complications. Interestingly, the way rural Bangladeshi women distinguished the symptoms of normal and complicated childbirth was somewhat related to a number of medical symptoms/signs of delivery. For instance, a recent ethnographic study in Bangladesh on women's understanding of birth (n=100) found that they were able to refer to several clinical signs of normal and complicated births. For example, to define complicated labour, they mentioned (a) prolonged labour pains without further progress, (b) membrane ruptured without labour pain, (c) *nari* (cord) comes out beforehand, and (d) becoming pregnant after a long interval of six years or more (20). Perhaps, a substantial proportion of our study women might have correctly recalled their recent childbirth circumstances and could remember whether they had an obstetrical complication or not during childbirth. However, some studies have noted poor agreement between women's reporting complicated delivery and confirmed medical diagnosis of obstetric complications (28,29).

Whether negative experiences of childbirth resulting from obstetric complications can lead to alterations in healthcare-seeking behaviour in subsequent pregnancies would be of interest to know. It would be particularly important to investigate the extent to which the quality of medical treatment and service might have contributed to the negative experience and whether this would have any effect on healthcare-seeking for subsequent pregnancies. Certainly, women who have severely uncomfortable pregnancy and childbirth experiences need to be flagged for special care and attention as they are more likely to develop postpartum depression as found in our study. These women need further evaluation of their psychological health, and simple interventions can be provided by trained lay village health workers to alleviate their low mood in this crucial period of their life. For example, modified cognitive behavioural therapy by female health workers has been found to be effective in reducing maternal depression in rural Pakistan (30).

### Limitations

A potential limitation of the study was that we could not absolutely confirm that women in the normal childbirth group with home-births had completely normal deliveries. It is possible that some of these women might have experienced clinical problems but were unable to access the hospital due to fami-lial, social and/or economic reasons. However, we believe that this did not impact significantly on our findings because of the study design and stringent methodology used in recruiting women for the study.

### Conclusions

Medically-defined obstetric complications were associated with a negative experience of childbirth. Women's childbirth experiences can provide important information for identifying the probable cases of postnatal depression.

## ACKNOWLEDGEMENTS

This study (Protocol No. 2005-039) was funded by the United States Agency for International Development (USAID), Washington, DC, through the Johns Hopkins School of Public Health (Grant No. GHS-A00-0300019-00). icddr,b acknowledges with gratitude the commitment of USAID to icddr,b's research efforts.
